# Supplementary material for: Radio Communications on Family Planning: Case of West Africa
Source: Int J Environ Res Public Health. 2022 Apr 11;19(8):4577. doi: 10.3390/ijerph19084577 (PMC9028430; doi:10.3390/ijerph19084577)
Supplement: Supplementary file 1 [file ijerph-19-04577-s001.zip › ijerph-1662294-supplementary.pdf]

SUPPLEMENTARY MATERIAL

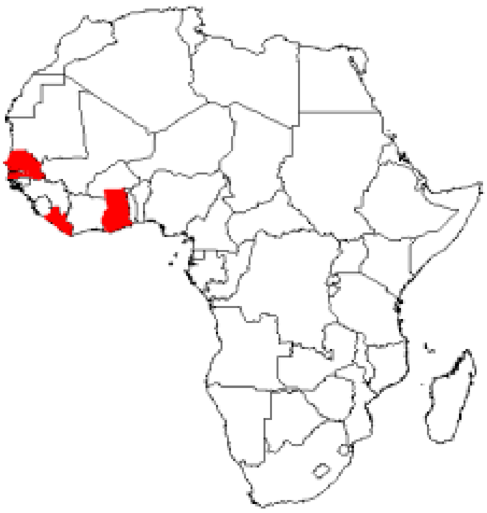

Figure S1. Study countries: Senegal (**Left**), Liberia (**Middle**), Ghana (**Right**).

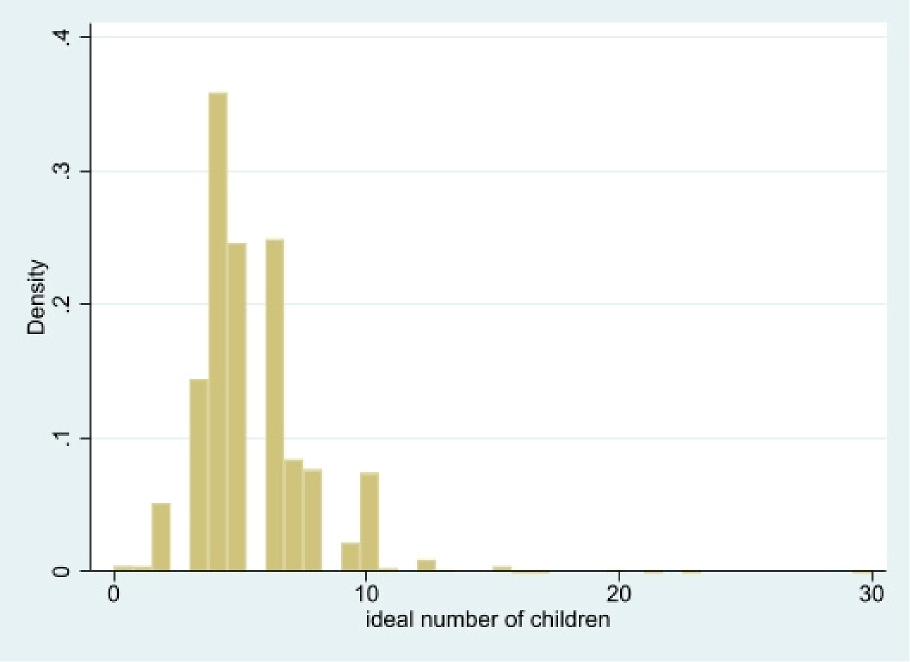

Figure S2. Dependent variable histogram.

```
. imb agen partage ipvcases specialdist literacy, treatment(trt)
(using the scott break method for L1 distance)

Multivariate L1 distance: .47719445

Univariate imbalance:

                   L1     mean      min      25%      50%      75%      max
       agen    .08006   .98418        0        1        1        1        0
    partage    .08755  -4.0065        0        0       -1       -4        0
   ipvcases    .11597  -.45311        0        0        0       -1        0
specialdist    .05768   .05768        0        0        0        1        0
   literacy     .1369    .1369        0        0        0        1        0
```

Figure S3. Initial imbalance in covariates (Stata output).

```
. cem agen partage ipvcases specialdist literacy, treatment(trt)

Matching Summary:
-----------------
Number of strata: 1674
Number of matched strata: 875

              0     1
      All  5400  6194
  Matched  4840  5492
Unmatched   560   702


Multivariate L1 distance: .36109327

Univariate imbalance:

                   L1     mean      min      25%      50%      75%      max
       agen    .04139   .04958        0        0        0        0        0
    partage    .03268  -.05651        0        0        0        0        .
   ipvcases   3.2e-15  8.7e-15        0        0        0        0        0
specialdist   6.9e-15  2.1e-15        0        0        0        0        0
   literacy   6.2e-15  2.8e-15        0        0        0        0        0
```

Figure S4. Automated CEM summary (Stata output).

```
. cem agen partage ipvcases specialdist literacy, treatment(trt) k2k
(using the scott break method for imbalance)

Matching Summary:
-----------------
Number of strata: 1674
Number of matched strata: 875

             0     1
      All  5400  6194
  Matched  3776  3776
Unmatched  1624  2418


Multivariate L1 distance: .32415254

Univariate imbalance:

                 L1     mean      min      25%      50%      75%      max
       agen  .04105   .06594        0        0        1        0        0
    partage  .01933  -.04052        0        0        0        0        .
   ipvcases       0        0        0        0        0        0        0
specialdist       0        0        0        0        0        0        0
   literacy       0        0        0        0        0        0        0
```

Figure S5. K-to-K matching summary (Stata output).

```
. regress wantnumchild trt [iweight = cem_weights]

      Source |       SS           df       MS      Number of obs   =     9,479
-------------+----------------------------------   F(1, 9477)      =     57.62
       Model |  272.776119         1  272.776119   Prob > F        =    0.0000
    Residual |  44863.7479     9,477  4.73396095   R-squared       =    0.0060
-------------+----------------------------------   Adj R-squared   =    0.0060
       Total |   45136.524     9,478   4.7622414   Root MSE        =    2.1757

------------------------------------------------------------------------------
wantnumchild | Coefficient  Std. err.      t    P>|t|     [95% conf. interval]
-------------+----------------------------------------------------------------
         trt |  -.3408973   .0449076    -7.59   0.000    -.4289259   -.2528687
       _cons |   5.392022   .0332701   162.07   0.000     5.326805    5.457238
------------------------------------------------------------------------------
```

Figure S6. SATT estimate/univariate regression results (Stata output).

```
. regress wantnumchild trt currentuse childdeaths fpdesire [iweight = cem_weights]

      Source |       SS           df       MS      Number of obs   =     9,478
-------------+----------------------------------   F(4, 9473)      =    168.66
       Model |  3000.60397         4  750.150991   Prob > F        =    0.0000
    Residual |  42134.4681     9,473  4.44784842   R-squared       =    0.0665
-------------+----------------------------------   Adj R-squared   =    0.0661
       Total |   45135.072     9,477   4.7625907   Root MSE        =    2.1089

------------------------------------------------------------------------------
wantnumchild | Coefficient  Std. err.      t    P>|t|     [95% conf. interval]
-------------+----------------------------------------------------------------
         trt |  -.3365893   .0435672    -7.73   0.000    -.4219904   -.2511883
  currentuse |  -.3461172   .0479247    -7.22   0.000    -.4400598   -.2521745
 childdeaths |    .580221   .0258323    22.46   0.000     .5295842    .6308578
    fpdesire |  -.4705135   .0467973   -10.05   0.000    -.5622463   -.3787807
       _cons |   5.416826   .0381277   142.07   0.000     5.342087    5.491564
------------------------------------------------------------------------------
```

Figure S7. Multiple linear regression results (Stata output).
